# Supplementary material for: Comparative Analysis of Serum N-Glycosylation in Endometriosis and Gynecologic Cancers
Source: Int J Mol Sci. 2025 Apr 25;26(9):4105. doi: 10.3390/ijms26094105 (PMC12071406; doi:10.3390/ijms26094105)

Supplementary

Comparative Analysis of Serum N-glycosylation in Endometriosis and Gynecologic Cancers

Róbert Pásztor[1,2], Béla Viskolcz[1], Csaba Oláh[2] and Csaba Váradi[1*]

[1] Institute of Chemistry, Faculty of Materials Science and Engineering, University of Miskolc, Miskolc, Hungary 3515

[2] Borsod-Abaúj-Zemplén County Center Hospital and University Teaching Hospital, Department of Neurosurgery, Miskolc, Hungary 3526

* Correspondence: csaba.varadi@uni-miskolc.hu; Tel.: +30-894-7730 (Cs.V.)

Supplementary Table S1. : Kruskall-Wallis test of serum N-glycan ratios in in Healthy control, Myoma uteri, Endometriosis and Cervix carcinoma patients by UPLC-HILIC-FLR

	**Control**	sdev	**Myoma uteri**	sdev	**Endometriosis**	sdev	**Cervix carcinoma**	sdev	p value
FA2	3,88	2,07	3,20	1,49	2,85	0,92	4,42	1,59	0,00
M5	0,83	0,24	0,70	0,23	0,72	0,21	0,59	0,12	0,00
FA2B	1,14	0,51	1,07	0,48	0,79	0,30	1,23	0,45	0,00
FA2(6)G1	3,53	0,95	3,26	0,74	3,47	0,75	4,02	1,20	0,09
FA2(3)G1	1,61	0,53	1,55	0,38	1,88	0,57	2,08	0,62	0,01
FA2BG1	1,34	0,36	1,34	0,32	1,19	0,33	1,44	0,42	0,10
M6	0,91	0,30	0,99	0,25	1,08	0,24	1,01	0,17	0,19
A2G2	0,72	0,13	0,88	0,13	0,88	0,12	1,02	0,16	0,00
FA2G2	2,98	0,83	2,96	0,90	3,67	1,13	3,30	1,13	0,13
FA2BG2	1,02	0,18	1,07	0,17	1,06	0,18	1,06	0,21	0,81
FA2G1S1	0,80	0,21	0,70	0,10	0,79	0,20	0,77	0,16	0,15
A2G2S1	10,30	0,96	11,84	0,74	11,27	0,76	12,34	1,51	0,00
FA2G2S1	4,59	0,73	4,18	0,88	5,00	1,01	4,46	1,15	0,03
A2G2S2 (1)	5,93	1,26	5,52	0,99	5,77	0,76	4,94	0,89	0,01
A2G2S2 (2)	33,80	3,05	35,88	2,95	33,21	3,14	33,13	3,98	0,03
FA2G2S2	5,35	1,42	5,12	0,64	5,08	0,81	5,20	1,05	0,99
FA2BG2S2	2,29	1,08	1,82	0,74	1,82	0,56	1,94	0,82	0,23
A2BG3S2	1,69	0,43	1,77	0,39	1,88	0,54	1,74	0,41	0,75
A3G3S2	1,46	0,36	1,64	0,35	1,53	0,35	1,62	0,33	0,32
FA3G3S2	1,19	0,32	1,09	0,27	1,25	0,20	1,22	0,31	0,25
A3G3S3 (1)	0,40	0,09	0,39	0,04	0,40	0,10	0,40	0,09	0,98
A3G3S3 (2)	6,25	1,78	5,80	1,38	6,29	1,90	4,90	1,25	0,03
A3G3S3 (3)	0,34	0,13	0,27	0,07	0,33	0,10	0,26	0,09	0,01
FA3G3S3 (1)	0,60	0,29	0,55	0,13	0,60	0,29	0,51	0,19	0,49
A3G3S3 (4)	1,79	0,60	1,90	0,63	1,73	0,49	1,69	0,45	0,54
FA3G3S3 (2)	2,89	1,08	2,57	1,30	2,73	1,31	2,81	1,46	0,88
FA3G3S3 (3)	0,90	0,17	0,78	0,12	0,96	0,24	0,76	0,15	0,00
A4G4S3	0,35	0,07	0,29	0,10	0,36	0,12	0,30	0,11	0,05
A4G4S4 (1)	0,50	0,21	0,33	0,11	0,60	0,23	0,33	0,15	0,00
A4G4S4 (2)	0,63	0,14	0,53	0,15	0,80	0,27	0,52	0,09	0,00

Supplementary Figure S1.: The importance of individual glycans in the separation of disease groups by LDA

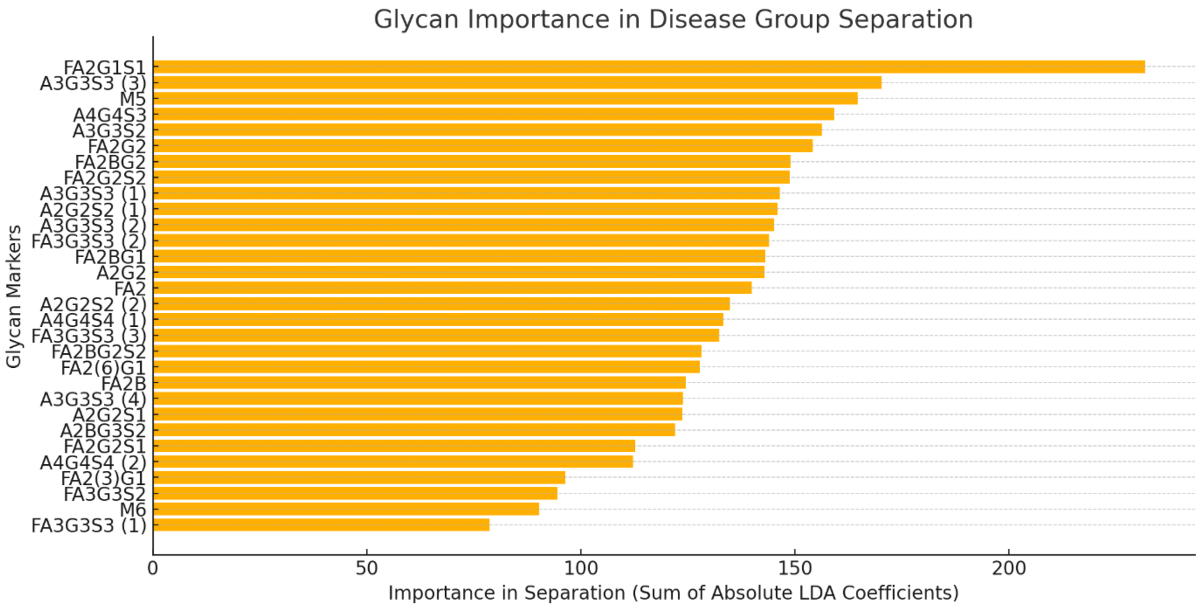

Supplementary Figure S2.: ROC curve analysis of most predictive glycan structures in the classification of control vs disease

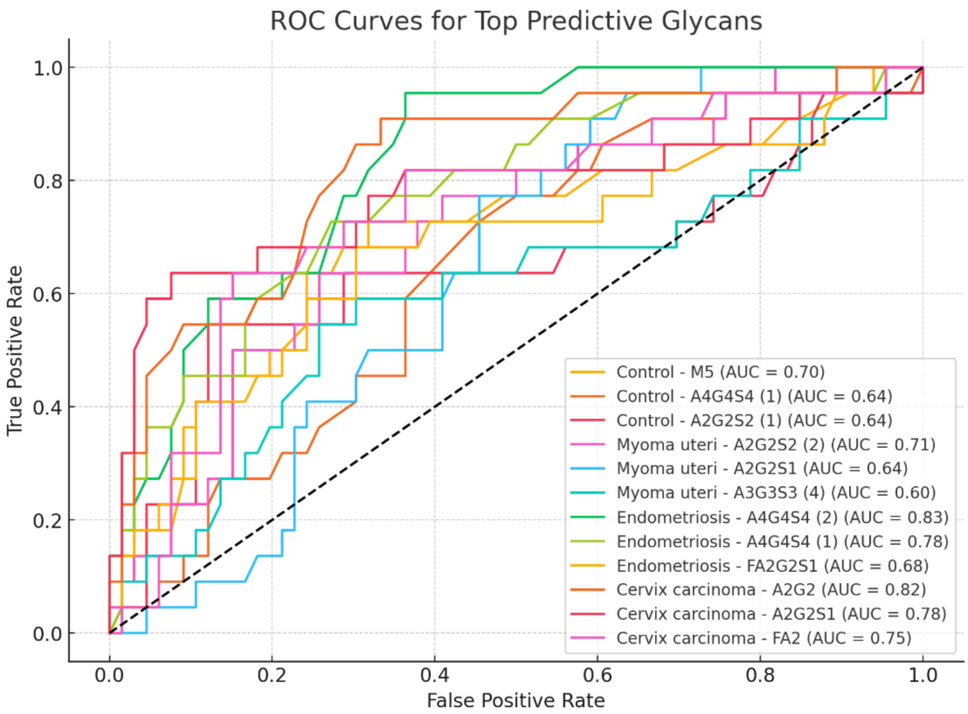

Supplement: Supplementary file 1 [file ijms-26-04105-s001.zip › ijms-3555570-supplementary.pdf]
